# Supplementary material for: Urban heat islands in China enhanced by haze pollution
Source: Nat Commun. 2016 Aug 23;7:12509. doi: 10.1038/ncomms12509 (PMC4996972; doi:10.1038/ncomms12509)
Supplement: Supplementary Information — Supplementary Table 1 [file ncomms12509-s1.pdf]

Supplementary Table 1: Coordinates and names of cities in Figure 1a.

Number	City, Province	Latitude (°)	Longitude (°)
1	Beijing	39.90	116.40
2	Tianjin	39.13	117.16
3	Shanghai	31.22	121.46
4	Nanjing, Jiangsu	32.05	118.77
5	Hangzhou, Zhejiang	30.29	120.16
6	Wuhan, Hubei	30.59	114.27
7	Zhengzhou, Henan	34.77	113.65
8	Hefei, Anhui	31.84	117.28
9	Jinan, Shandong	36.66	116.98
10	Shijiazhuang, Hebei	38.05	114.48
11	Taiyuan, Shanxi	37.87	112.57
12	Shenyang, Liaoning	41.81	123.43
13	Harbin, Heilongjiang	45.75	126.67
14	Changchun, Jilin	43.91	125.31
15	Xi'an, Shannxi	34.27	108.94
16	Haikou, Hainan	20.03	110.35
17	Guiyang, Guizhou	26.58	106.71
18	Hohhot, Inner Mongolia	40.84	111.69
19	Baotou, Inner Mongolia	40.65	109.82
20	Nanning, Guangxi	22.85	108.32
21	Yueyang, Hunan	29.37	113.13
22	Urumqi, Xinjiang	43.87	87.56
23	Hami, Xinjiang	42.83	93.51
24	Korla, Xinjiang	41.75	86.16
25	Chengdu, Sichuan	30.67	104.07
26	Meishan, Sichuan	30.04	103.83
27	Jieyang, Guangdong	23.54	116.37
28	Nanchang, Jiangxi	28.66	115.91
29	Xining, Qinghai	36.61	101.80
30	Yuxi, Yunnan	25.50	103.80
31	Yinchuan, Ningxia	38.46	106.27
32	Zhangye, Gansu	38.93	100.46
33	Lhasa, Tibet	29.65	91.13
34	Zhangzhou, Fujian	24.52	117.67
35	Jiamusi, Heilongjiang	46.80	130.36
36	Hulunbir, Inner Mongolia	49.22	119.73
37	Delhi, Qinghai	37.36	97.36
38	Hotan, Xinjiang	37.12	79.92
39	Qingyuan, Guangdong	23.72	113.03
